# A Statistical Model of the International Spread of Wild Poliovirus in Africa Used to Predict and Prevent Outbreaks

**DOI:** 10.1371/journal.pmed.1001109

**Published:** 2011-10-18

**Authors:** Kathleen M. O'Reilly, Claire Chauvin, R. Bruce Aylward, Chris Maher, Sam Okiror, Chris Wolff, Deo Nshmirimana, Christl A. Donnelly, Nicholas C. Grassly

**Affiliations:** 1 Medical Research Council Centre for Outbreak Analysis and Modelling, Department of Infectious Disease Epidemiology, School of Public Health, Imperial College London, London, United Kingdom; 2 Global Polio Eradication Initiative, World Health Organization, Geneva, Switzerland; 3 Immunizations and Vaccine Development, World Health Organization, Brazzaville, Republic of the Congo; The National Institute for Public Health and the Environment, Netherlands

## Abstract

**Background:**

Outbreaks of poliomyelitis in African countries that were previously free of wild-type poliovirus cost the Global Polio Eradication Initiative US$850 million during 2003–2009, and have limited the ability of the program to focus on endemic countries. A quantitative understanding of the factors that predict the distribution and timing of outbreaks will enable their prevention and facilitate the completion of global eradication.

**Methods and Findings:**

Children with poliomyelitis in Africa from 1 January 2003 to 31 December 2010 were identified through routine surveillance of cases of acute flaccid paralysis, and separate outbreaks associated with importation of wild-type poliovirus were defined using the genetic relatedness of these viruses in the VP1/2A region. Potential explanatory variables were examined for their association with the number, size, and duration of poliomyelitis outbreaks in 6-mo periods using multivariable regression analysis. The predictive ability of 6-mo-ahead forecasts of poliomyelitis outbreaks in each country based on the regression model was assessed. A total of 142 genetically distinct outbreaks of poliomyelitis were recorded in 25 African countries, resulting in 1–228 cases (median of two cases). The estimated number of people arriving from infected countries and <5-y childhood mortality were independently associated with the number of outbreaks. Immunisation coverage based on the reported vaccination history of children with non-polio acute flaccid paralysis was associated with the duration and size of each outbreak, as well as the number of outbreaks. Six-month-ahead forecasts of the number of outbreaks in a country or region changed over time and had a predictive ability of 82%.

**Conclusions:**

Outbreaks of poliomyelitis resulted primarily from continued transmission in Nigeria and the poor immunisation status of populations in neighbouring countries. From 1 January 2010 to 30 June 2011, reduced transmission in Nigeria and increased incidence in reinfected countries in west and central Africa have changed the geographical risk of polio outbreaks, and will require careful immunisation planning to limit onward spread.

*Please see later in the article for the Editors' Summary*

## Abstract 2

Using outbreak data from 2003–2010, Kathleen O'Reilly and colleagues develop a statistical model of the spread of wild polioviruses in Africa that can predict polio outbreaks six months in advance.

## Editors' Summary

**Background:**

During the first half of the 20th century, polio (poliomyelitis) was one of the most feared infectious diseases in industrialized countries, paralyzing thousands of young children every year. The virus that causes polio enters the human body through ingestion of contaminated water or food, multiplies in the gut, and is shed through the feces (stool) into the environment, where it spreads rapidly if sanitation or personal hygiene is poor. Most people infected with poliovirus have no symptoms, but about one in 200 infected people develop paralytic polio, in which poliovirus invades and destroys the nerve cells that control the arm and leg muscles, leading to acute flaccid paralysis (AFP; limb paralysis). In the worst cases, poliovirus paralyzes the muscles involved in breathing, which can be fatal unless patients are helped to breathe with an “iron lung” or ventilator. There is no cure for paralytic polio, and although AFP usually lasts less than two weeks, some patients never regain full use of their limbs.

**Why Was This Study Done?:**

From 1955 onwards, routine polio vaccination rapidly reduced or eliminated wild polio (polio occurring through natural infection) in developed countries, but the disease remained common in developing countries. Consequently, in 1988, the Global Polio Eradication Initiative was launched. Between 1988 and 2009, routine vaccination and supplementary immunization activities (additional doses of polio vaccine given to all young children on national immunization days) reduced the annual number of children paralyzed by polio from 350,000 to about 1,600 and the number of countries where polio is endemic (always present) from 125 to four. Unfortunately, continued circulation of wild polioviruses in Nigeria and India resulted in reinfection of 19 African countries in 2009 and re-establishment of polio transmission in four countries. A better understanding of the factors that affect the distribution and timing of wild polio outbreaks might help experts prevent such outbreaks and could facilitate global polio eradication. Here, the researchers develop a statistical model of the spread of wild polioviruses in Africa and assess its ability to predict polio outbreaks in individual African countries.

**What Did the Researchers Do and Find?:**

The researchers used routine AFP surveillance to identify children who developed polio in Africa between 2003 and 2010. They determined whether each case was associated with the importation of wild poliovirus based on genetic analysis the polioviruses and then used “multivariable regression analysis” to identify factors associated with the number, size, and duration of polio outbreaks. During the study period, 142 genetically distinct polio outbreaks (involving one to 228 cases) were recorded in 25 African countries, with the average number of outbreaks in each country declining with reduced population movements from each infected country. The estimated number of people migrating into a country from an infected country was associated with the number of outbreaks in that country. Thus, countries with a high rate of immigration from Nigeria and other countries where polio is still endemic had more polio outbreaks than countries with less immigration from these countries. A country's mortality rate for children under 5 years of age (an indicator of sanitary conditions and access to health care) was also associated with the number of outbreaks, and immunization coverage was associated with the size, duration, and number of outbreaks. Finally, in 82% of instances, for a randomly selected country where an outbreak was observed, the statistical model predicted six months ahead of time more outbreaks for that country than for any randomly selected country where there were no outbreaks. That is, the model's predictive ability was 82%.

**What Do These Findings Mean?:**

These findings indicate that outbreaks of polio in Africa over the study period resulted mainly from continued transmission in Nigeria and other countries that reported polio cases and from poor immunization status. They also highlight how the geographical risk of polio is changing over time in Africa. Importantly, the risk factors included by the researchers in their statistical model are sufficient to describe the scale and geographical distribution of polio outbreaks in Africa six months in advance with a high predictive ability. Although the accuracy of the predictions made by the model is limited by the structure of the model and by the data fed into it, the information provided by this and other predictive models should help the Global Polio Eradication Initiative plan its future immunization and surveillance campaigns and should facilitate the elimination of polio from Africa.

**Additional Information:**

Please access these websites via the online version of this summary at http://dx.doi.org/10.1371/journal.pmed.1001109.

- The Global Polio Eradication Initiative provides information about polio and about global efforts to eradicate the disease; its website includes links to videos about global polio elimination efforts
- The World Health Organization provides information about polio and attempts to eradicate the disease (in several languages)
- The US Centers for Disease Control and Prevention provides information about polio vaccination
- The UK National Health Service Choices website has information on polio
- MedlinePlus provides links to more resources on polio (in English and Spanish)
- Personal stories about polio are available through the British Polio Fellowship Heritage Project; the National Museum of American History Whatever happened to polio? website includes an archive of polio-related pictures

## Introduction

The success of the oral poliovirus vaccine (OPV) in eliminating polio in the Americas led to a commitment by the governments of the world to eradicate polio at the World Health Assembly in 1988. Since that time, widespread use of OPV has reduced the number of children paralysed by polio from an estimated 350,000 in 1988 to just 1,606 in 2009 [1]. Early estimates of incidence in the African continent are affected by substantial under-reporting; however, 4,546 cases were reported in Africa in 1988, and by 2009 this was reduced to 693 cases [2]. This decrease in reported cases has been accompanied by a marked reduction in the geographic extent of endemic areas, such that by 2006 just four countries had yet to interrupt indigenous wild-type poliovirus transmission worldwide, and in Africa only Nigeria was endemic for polio. Transmission has been persistent in those countries, and onward spread to previously polio-free areas has presented significant challenges to the Global Polio Eradication Initiative (GPEI).

Continued circulation of wild polioviruses in Nigeria and India led to reinfection of 19 African countries in 2009 alone [1], and cost the GPEI nearly US$150 million that year. Four of the reinfected countries (Angola, Chad, the Democratic Republic of the Congo [DRC], and Sudan) were considered to have re-established poliovirus transmission, requiring eradication efforts and resources proportionate to those in the endemic countries [3]. Efforts to control poliovirus in these reinfected countries have continued in 2010 with some success, notably in Sudan [4]. Despite the reported successes, importation of poliovirus to polio-free countries has continued. For example, the importation of poliovirus to the Congo in 2010 caused the first outbreak of poliomyelitis in that country in 10 y, resulting in approximately 554 cases [5]. Development of the GPEI Strategic Plan 2010–2012 builds upon lessons learnt during previous experiences, and includes novel approaches to interrupting poliovirus transmission and preventing outbreaks [6]. The analysis presented here is part of the framework within the Strategic Plan to use statistical and mathematical models to aid decision making.

The risk of an importation of wild-type poliovirus into a country (where an importation is a case of poliomyelitis for which the associated poliovirus in a stool sample has a genetic sequence distinct from cases already indentified in that country) is likely to be influenced by exposure to poliovirus circulating in other countries and the immunity profile of the local population [7]. Environmental and demographic characteristics related to crowding, population movement, and sanitation are also likely to play a role. The relative importance of different population characteristics, and the effect of routine immunisation and supplementary immunisation activities (SIAs) on the number and size of outbreaks experienced by a country, have not been formally assessed or quantified. Here we use detailed surveillance data from 56 countries in Africa to identify the most important risk factors for an outbreak, and to identify those variables associated with a reduced number of outbreaks and rapid interruption of the imported wild virus. Furthermore, we examine the predictive ability of a statistical model based on these variables to identify countries and regions at risk of an outbreak 6 mo ahead of time.

## Methods

### Description of the Data

Outbreaks of poliomyelitis that occurred on the African continent between 1 January 2003 and 31 December 2010 were identified through routine surveillance of cases of acute flaccid paralysis (AFP). All countries reported cases of AFP through a network of health care workers to achieve a minimum surveillance standard of one non-polio AFP case per 100,000 children under 15 y of age. A case of poliomyelitis was confirmed when wild-type poliovirus was isolated from at least one of two stool samples collected from each child with AFP. Outbreaks were defined on the basis of their genetic identity and their location. The serotype, genotype, and cluster were investigated by the US Centers for Disease Control and Prevention and the World Health Organization (WHO) to define epidemiologically linked cases, where the presence of different capsid proteins defined the serotype [8], >85% nucleotide similarity of the VP1/2A interval defined the genotype [9], and independent transmission pathways determined by evolutionary divergence defined the cluster [10],[11]. An outbreak was defined as one or more cases of poliomyelitis within a country that had never previously reported other cases genetically linked to that cluster. Even a single child with AFP and wild-type poliovirus in stool samples was considered an outbreak if associated with a unique genetic cluster of poliovirus, because each reported case of paralysis is likely to be associated with several hundred or more asymptomatic or unreported infections [12]. Only outbreaks of wild-type virus were considered; vaccine-derived poliovirus outbreaks were excluded from the analysis.

Spread of a virus cluster across a country border resulted in the recording of the associated cases as a new outbreak in the newly affected country. The DRC, Ethiopia, and Sudan were split into regions because of their size and because immunisation activities were often carried out in discrete regions smaller than the size of the country. Additionally, the reported number of OPV doses varied considerably between these regions, as a result of the different vaccination schedules, coverage of the population through routine vaccination services, and socio-economic factors (see Text S1). In these countries, spread of a cluster from one region to the next was regarded as a new outbreak.

Explanatory variables were generated at a country level or a regional level (DRC, Ethiopia, and Sudan) and grouped into 6-mo intervals to account for changes over time. Explanatory variables that were tested in the model included those related to population immunity and underlying characteristics such as population density, childhood mortality, and indices of poverty that may influence outbreak susceptibility. Measures of population immunity included country-specific estimates of routine coverage from WHO/United Nations Children's Fund (the proportion of children who have received three or more doses of trivalent OPV by their second birthday through routine immunisation), and the proportion of children aged 0–4 y old who received three or more doses of OPV of any type, obtained from non-polio AFP records. The reported vaccination status of children under 5 y of age who had AFP and from whom two adequate stool samples were collected but without any poliovirus isolated (referred to as non-polio AFP cases) was assumed to reflect the vaccination status of the rest of the community, based on previous research [13],[14].

A variable termed “poliovirus exposure” (λ) was used to describe exposure in a given country to wild-type poliovirus due to movement of people from other countries with circulating poliovirus. Four data sources approximating population movement between African countries and from the remaining endemic countries (Afghanistan, India, Nigeria, and Pakistan) were examined for their association with poliomyelitis outbreaks. These sources describe the number of passengers on flights in 2008 [15], permanent migrants in 2001 [16], tourists in 2004 [17], and the population size of each pair of countries scaled by the physical distances between their capital cities (also known as a gravity model [18]; see Text S1). Poliovirus exposure in a given 6-mo period *t* experienced by country *j* from country *i* was calculated as
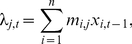
(1)where 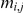 is the reported number of individuals in each dataset who moved from *i* to *j* (or other approximation), 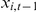 is the number of poliomyelitis cases reported in country *i* during the preceding 6-mo period, and *n* is the number of countries. In other words, the incidence in country *i* at *t*−1 was multiplied by the reported number of movements from *i* to *j*, and this value was summed for all countries in Africa to obtain the total poliovirus exposure in country *j*. In addition, the contribution of specific regions or countries to poliovirus exposure was examined by calculating this measure separately for exposure from Nigeria, from other countries in Africa, and from Afghanistan, India, and Pakistan (the remaining endemic countries outside of Africa). Poliovirus exposure from the previous 7–12 and 13–18 mo was also examined to explore time-varying effects. Six-month intervals were chosen for this analysis to maximise temporal resolution but at the same time ensure that estimates of vaccine coverage were based on a sufficient number of children with AFP.

### Statistical Analysis

Regression models were used to identify factors associated with the number of wild poliovirus outbreaks reported by a country or region for every 6 mo of the study period, the duration of these outbreaks, and their size. A Poisson mixed effects regression model was used to identify factors associated with the number of outbreaks from 1 July 2004 to 31 December 2010. A mixed effects model structure was used to account for multiple observations per country. A Poisson log-normal mixed effects model, which accounts for overdispersion of the number of outbreaks, was also tested, and the predictive ability of both models compared [19]. Outbreaks that began prior to 1 July 2004 were excluded from the analysis as there were no data on population immunity or poliovirus exposure prior to 18 mo before this time period (January 2003 was the first available time point for the data). Explanatory variables significant (*p*<0.2) at a univariable level were incrementally added to the multivariable model if the Akaike's information criterion (AIC) reduced significantly in value, known as a stepwise addition approach [19]. Interactions were tested between variables in the multivariable model, and all variables were tested for their inclusion and interactions with variables in the final model. 95% confidence intervals (CIs) for the model fit were calculated from the standard error (SE) of the fixed effects. Models were implemented in the software STATA (version 11.0).

The regression analysis of the number of outbreaks included only variables from previous time periods, and so it was possible to forecast the number of outbreaks expected in a country 6 mo ahead of time. The predictive accuracy of these forecasts was estimated using prospective sampling, where the longitudinal data used to estimate regression model parameters were truncated and forecasts of the next 6 mo compared with observed data [20]. Six-month-ahead predictions were made for 1 July–31 December 2007 onwards. For each model prediction, the limits of the prediction interval were obtained from the 2.5 and 97.5 percentiles of the negative binomial distribution, with mean equal to the model prediction and variance equal to the sum of the variance of the model prediction and the variance around the expected value associated with a Poisson distribution [21]. Receiver operator characteristic analysis was used to assess the accuracy of the predictions, where the area under the curve (AUC) was estimated using a binormal parametric model [22]. The estimated value of the AUC is equivalent to the probability that a randomly drawn country where an outbreak was detected had a greater predictive value than a randomly drawn country where no outbreak was detected. The AUC value should at least be greater than 0.50 (which would indicate that the model is no better than random), and values above 0.80 indicate a good predictive ability [23],[24]. The Poisson model and the Poisson log-normal model were tested for their predictive ability. By way of comparison, the number of outbreaks per country in the previous 3 y was used as a measure of historical propensity for outbreaks and used to predict whether an outbreak would occur in the current 6 mo (other time periods were also explored; see Text S1). To predict likely outbreaks in the period 1 January–30 June 2011, data on outbreaks from the period 1 July 2004 to 31 December 2010 were used in the regression model to estimate coefficients, and these coefficients were combined with predictors as measured in the preceding 6 mo.

Factors associated with the duration of outbreaks were modelled using a Cox proportional-hazards model with censoring to account for ongoing outbreaks [25]. The duration of an outbreak was defined as the time (in days) from the first to the last case of an outbreak. Outbreaks were declared over when there were no epidemiologically linked cases in the country or region for at least 6 mo after the last case; otherwise, outbreaks were assumed to be ongoing. Factors affecting the size of an outbreak were investigated using a censored negative binomial generalised linear model, as the variance was larger than the mean size of outbreaks [26]. For analyses of outbreak duration and size, those that began between 1 July 2003 and 31 June 2010 were included in the analysis.

## Results

### Distribution of the Size and Geographic Extent of Poliomyelitis Outbreaks in Africa

**Figure 1:**
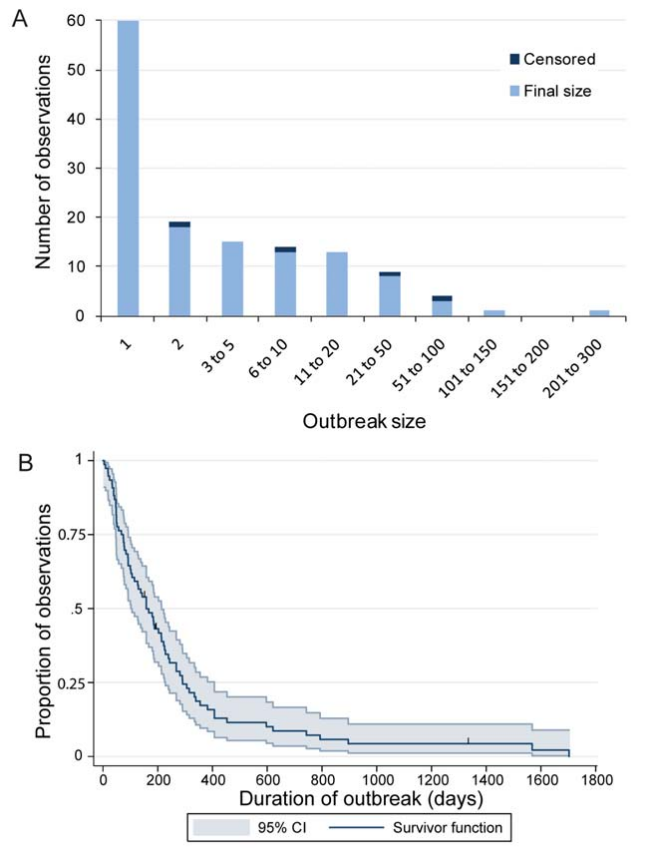
Distribution of the size and duration of outbreaks in Africa 2003–2010. (A) Size of outbreaks. (B) Duration of outbreaks. Where no epidemiologically linked cases have been detected in the last 6 mo the final size is reported. If cases have been recently (1 July–31 December 2010) detected, the size and duration are censored. All censored outbreaks are denoted by the blue tick marks in the Kaplan-Meier curve (B).

Between 1 July 2003 and 31 December 2010 there were 25 countries in Africa that reported an outbreak of polio, corresponding to 137 outbreaks. When these outbreaks were further divided into the regions within Ethiopia, Sudan, and DRC, this corresponded to 142 outbreaks; the rest of the analysis refers to these data. The median size of outbreaks was two cases (range 1–228; Figure 1A), and the median duration of outbreaks was 39 d when not accounting for censoring. Four outbreaks with cases reported after 1 July 2010 were classified as ongoing outbreaks that were censored. Eighty-one outbreaks (57%) consisted of more than one case of poliomyelitis, and the median duration of these outbreaks was 158 d. After correcting for censoring, the Kaplan-Meier estimates of the median duration of the 81 multiple-case outbreaks was 163 d (Figure 1B).

**Figure 2:**
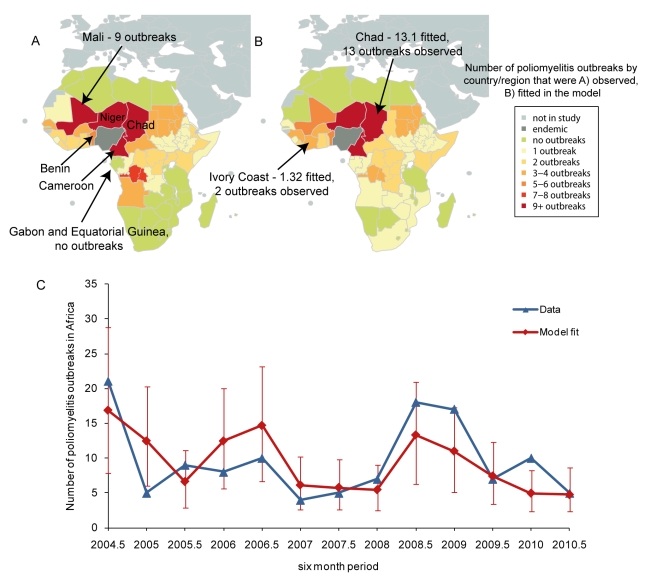
Distribution of the risk of poliomyelitis outbreaks in Africa. (A) The number of poliomyelitis outbreaks reported for each country in Africa between 1 July 2004 and 31 December 2010. (B) The expected number of poliomyelitis outbreaks for each country in Africa based on the fit of the Poisson mixed effects model. (C) The temporal fit of the Poisson mixed effects model, where error bars show the 95% CIs, and the reported number of outbreaks for each 6-mo period.

The average number of outbreaks experienced by each country or region declined with physical distance from Nigeria (Figure 2A). Cameroon, Chad, and Niger, all bordering Nigeria, reported nine or more outbreaks each during the study period, and Niger reported 33 outbreaks, of which 21 were single cases. Benin, also bordering Nigeria, reported seven outbreaks during the study period. Further away from Nigeria, Mali, Burkina Faso, and the Central African Republic reported five or more outbreaks each. Eight countries—Botswana, Burundi, the Congo, Eritrea, Mauritania, Namibia, Sierra Leone, and Somalia—reported only one outbreak during the study period (further details are given in Text S1). South Sudan, south DRC, northeast Ethiopia, and southeast Ethiopia reported one outbreak each during the study period.

### Factors Associated with the Risk of Outbreaks

Fifteen explanatory variables were associated (*p*<0.2) with the number of outbreaks in the univariable analysis, and were used to develop the multivariable model (Table 1). The final Poisson mixed effects regression model retained five explanatory variables (Table 2). These included the measure of exposure to wild-type polioviruses based on the distribution of children with poliomyelitis in the preceding 6 mo and migration from affected countries. Poliovirus exposure measured by the number of permanent migrants moving between countries was found to provide a better fit to the data than exposure terms based on the movement of tourists, flight data, or physical distance. If exposure was higher in the previous 6 mo than 18 mo ago, there was an additional increase in risk. Countries physically bordering Nigeria (Benin, Chad, Niger, and Cameroon), with poor immunisation coverage (based on AFP data) and high child mortality also had an increased risk of an outbreak.

**Table 1:** Univariate analysis of variables associated with the number of poliomyelitis outbreaks in Africa from 1 January 2004 to 31 December 2010.

Description	Data Source	Incidence Risk Ratio			*p*-Value
		Median	2.5th Percentile CI	97.5th Percentile CI	
**Estimates of poliomyelitis exposure**					
Using data on international migrants from all African countries; 1 unit increase (when logged)	[16]; WHO	1.61	1.36	1.87	<0.001
Using data on international migrants from Nigeria; 1 unit increase (when logged)	[16]; WHO	1.36	1.19	1.53	<0.001
Using data on international migrants from Asia; 1 unit increase (logged)	[16]; WHO	0.94	0.81	1.02	0.27
Using data on international migrants: exposure 6 mo ago from all African countries was higher than that 18 mo ago (versus lower)	[16]; WHO	2.67	1.80	3.91	<0.001
Using data on international tourism from Nigeria only; 1 unit increase (when logged)^a^	[17]; WHO	1.22	1.10	1.35	<0.001
Using distance between capital cities from all African countries; 1 unit increase (logged)	WHO	1.45	1.20	1.71	<0.001
Using distance between capital cities from Nigeria; 1 unit increase (logged)	WHO	1.43	1.13	1.58	<0.001
Country borders Nigeria (versus does not)	—	13.37	4.82	29.56	<0.001
**Estimates of population immunity**					
10% increase in the percentage of non-polio AFP cases reporting three or more doses of OPV	WHO	0.81	0.68	0.91	<0.001
10% increase in the percentage of children reporting three doses of the OPV through routine coverage	UN/WHO	0.85	0.72	0.99	0.03
Median number of OPV doses reported in non-polio AFP cases; 1 unit increase	WHO	0.85	0.71	1.03	0.1
10% increase in the percentage of non-polio AFP cases reporting no doses of OPV	WHO	1.26	0.98	1.62	0.06
**Other factors**					
10% increase in percentage of the population aged 0–14 y	[33]	5.62	2.20	14.33	<0.001
Country reported a <5-y mortality rate greater than 150 deaths per 1,000 at-risk population (versus lower)	UN	2.88	1.24	6.71	<0.001
10% increase in the percentage of the population below the poverty line (country definitions vary)	[33]	1.08	0.87	1.35	0.55
Density of population (people per km^2^); 1 unit increase	[33]	0.99	0.99	1.00	0.07
Population size; 1 unit increase (logged)	[33]	1.37	0.86	2.20	0.15
Number of AFP cases reported per country in children under 5 y; 1 unit increase	WHO	1.00	1.00	1.00	0.81
AFP rate (cases per 100,000 population aged 0–14 y); 1 unit increase	WHO	0.96	0.78	1.19	0.71

*n* = 622 from 56 countries.

a No tourists were reported from Nigeria for nine countries, all of which are comparatively small in population size. To include these countries in the analysis 0.5 was added to the number of tourists before multiplying by the incidence in Nigeria and log-transforming the data.

**Table 2:** Final multivariable Poisson mixed effects model describing variables associated with the number of poliomyelitis outbreaks in Africa from 1 January 2004 to 31 December 2010.

Description of Risk Factor	Incidence Risk Ratio			*p*-Value	Standard Deviation of Random Effect
	Median	2.5th Percentile CI	97.5th Percentile CI		
Poliomyelitis exposure from all African countries; 1 unit increase (when logged)	1.33	1.15	1.54	<0.001	N/A
Poliomyelitis exposure in previous 6 mo was higher than exposure 18 mo ago (versus lower)	1.91	1.27	2.87	0.002	N/A
Country borders Nigeria (versus does not)	5.39	2.69	10.78	<0.001	N/A
10% increase in the percentage of non-polio AFP cases reporting three or more doses of OPV	0.85	0.75	0.97	0.013	0.71
Country reported a <5-y mortality rate greater than 150 deaths per 1,000 at-risk population (versus lower)	2.3	1.3	4.08	0.004	N/A

*n* = 622 from 56 countries; AIC = 548.06.

N/A, not applicable.

Niger, Chad, and Cameroon reported more than nine outbreaks, and this was replicated in the model, although Mali had more outbreaks than expected (9 compared with 5.31; Figure 2B). In west Africa, there were generally slightly more reported outbreaks than expected from the model. For example, the Ivory Coast reported two outbreaks and the model expectation was 1.32 outbreaks. For Eritrea, Gabon, Equatorial Guinea, and countries south of DRC, which did not report outbreaks during the study, the model expectation was that each would have had at least one outbreak. Countries in north Africa (Algeria, Egypt, Libya, Morocco, and Tunisia) reported no outbreaks, which was similar to what was expected in the model. The temporal fit of the number of outbreaks showed a large number of outbreaks in the second halves of 2004 and 2008, and a reduction in outbreaks during 2007 and 2010 (Figure 2C). However, the large number of outbreaks observed from mid-2008 to 2009 and the ten outbreaks observed in 2010 were not replicated in the model. The 95% CI of the model fit included the reported number of outbreaks, but the CIs were wide, particularly when a large number of outbreaks was observed.

When routine vaccination coverage in the preceding 6 mo was included in the regression model instead of vaccine coverage based on the reports for non-polio AFP cases, the fit of the model worsened (AIC of the model was 553.82 compared to 548.06; see Text S1). However, routine coverage was correlated with the proportion of non-polio AFP cases reporting at least three doses of OPV (correlation coefficient 0.54, *p*<0.001; see Text S1).

### Forecasting the Number of Outbreaks in Africa

**Figure 3:**
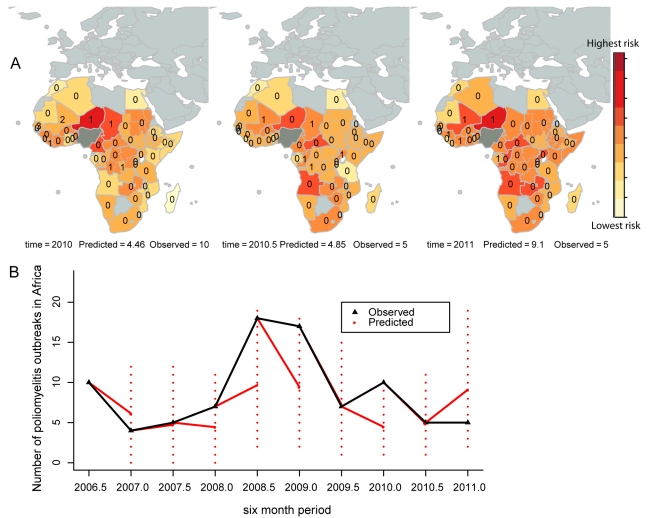
Six-month-ahead predictions and comparison to the observed number of outbreaks. (A) Predictions from 1 January 2010 to 30 June 2011 are illustrated from left to right, along with the observed number of outbreaks for the first and second halves of 2010. (B) The temporal predictions from 2007 (red lines) and the prediction intervals (dashed red lines) are illustrated. The observed number of outbreaks for each 6-mo period are overlaid (black lines). The predictive ability of the model was estimated to be 82%. Years (e.g., 2010) indicate the first half of the year (1 January–30 June); years plus 0.5 (e.g., 2010.5) indicate the second half of the year (1 July–31 December).

The AUC of the Poisson regression model was 0.82 (SE = 0.03), indicating a good predictive ability for the number of future outbreaks. In other words, 82% of the time for a randomly selected country (or region) and period where an outbreak was observed, the predicted number of outbreaks was greater than any other randomly selected country (or region) and period where no outbreak was observed. The Poisson log-normal mixed effects regression model had a reduced predictive ability of 0.77 (SE = 0.05), despite an improved fit to the data (AIC of the Poisson log-normal model was 528.39 compared to 548.06). Therefore, the rest of the analyses are based on results from the Poisson regression model. The model that used the number of outbreaks in the previous 3 y as a predictor for outbreaks in the current 6 mo had a predictive ability of 0.76 (SE = 0.04). The model of historical propensity can predict outbreaks only in countries that have previously reported an outbreak, whereas the Poisson regression model predicts the risk of an outbreak based upon values of the parameters and coefficients. Inspecting the predictive map for 2010 shows that Niger, Chad, and Cameroon were predicted to have the highest chance of an outbreak, but only Niger and Chad reported outbreaks within the 6-mo period (Figure 3A). Mali and Senegal reported two or more outbreaks but were predicted to have fewer outbreaks than either Niger or Chad. In the second half of 2010 the Congo and Uganda were predicted to have an increased risk when compared to the previous time period, and an outbreak was reported in both countries during this time. The predicted increase in risk in countries south of DRC (Angola, Zambia, and Rwanda, for example) in 2010 has not been associated with any reported outbreaks to date (although Angola has persistent transmission from a previous outbreak). The temporal trends illustrate that the model was able to predict the decrease in outbreaks in the first half of 2007, both halves of 2009, and the second half of 2010 when compared to previous time periods, and predicted the increase in outbreaks in the second halves of 2007 and 2008 (Figure 3B). For all time periods, the 95% prediction intervals include the observed number of outbreaks, and, with the exception of 2011, the prediction intervals decrease in size with the addition of more data.

Based on data prior to 1 January 2011, the predicted number of outbreaks in Africa from 1 January to 31 June 2011 was 9.10 (95% prediction interval = 2–19 outbreaks). The nine countries with the highest predicted risk were, starting with the highest, Niger, Angola, Mali, Central African Republic, the Congo, Uganda, Zambia, Rwanda, and Cameroon. As of 12 July 2011, five countries had reported outbreaks: Côte d'Ivoire, Guinea, Gabon, Mali, and Niger [27].

### Factors Associated with the Duration and Size of an Outbreak

Of the 137 outbreaks recorded prior to 1 July 2010, 133 (97%) were fully observed and the remainder were right censored (i.e., were regarded as ongoing outbreaks). The hazard ratio of a Cox proportional-hazards model represents the effect of a unit change in the explanatory variable on the frequency of the outcome, which in this case is the last case of an outbreak. Higher vaccination coverage in the 6 mo prior to an outbreak and sharing a border with Nigeria were both associated with shorter and smaller outbreaks (Tables 3 and 4). In addition, an annual AFP rate greater than two cases per 100,000 children under 15 y of age was associated with smaller outbreaks (Table 3).

**Table 3:** Final multivariable Cox proportional-hazards survival model describing variables associated with outbreak duration.

Description of Risk Factor	Hazard Ratio			*p*-Value
	Median	2.5th Percentile CI	97.5th Percentile CI	
10% increase in the percentage of non-polio AFP reporting three or more doses of OPV	1.14	1.03	1.26	0.011
Country borders Nigeria (versus does not)	1.58	1.11	2.24	0.010

*n* = 136 observations; log-likelihood = −532.25.

The hazard ratio represents the effect of a unit change in the explanatory variable on the frequency of the outcome, which in this case is the last case of an outbreak. If the hazard ratio for a variable is greater than 1.00, an increase in the variable results in an increase of the hazard. In other words, when the value of the variable increases, an outbreak is expected to be of a shorter duration.

**Table 4:** Final multivariable negative binomial model describing variables associated with the number of cases reported during an outbreak.

Variable	Incidence Risk Ratio			*p*-Value
	Median	2.5th Percentile CI	97.5th Percentile CI	
10% increase in the percentage of non-polio AFP cases reporting three or more doses of OPV	0.85	0.76	0.96	0.032
Country borders Nigeria (versus does not)	0.41	0.27	0.63	<0.001
AFP rate is greater than two cases per 100,000 population aged 0–14 y (versus less than)	0.49	0.30	0.80	<0.001

*n* = 135; log-likelihood = −385.7.

## Discussion

In this paper we demonstrate a clear link between vaccination coverage and population movement from endemic regions and the risk of outbreaks of poliomyelitis in Africa. Significant temporal and geographic variation in the number of reported outbreaks is explained by changes in population immunity and exposure to wild poliovirus imported by travellers from affected countries. Notably, a model incorporating population movement proportional to the number of permanent migrants was found to fit the data better than models based on physical distance, tourism, or flight data. The identified risk factors are sufficient to describe the scale and geographic distribution polio outbreaks in Africa 6 mo in advance with a predictive ability of 82%. Given the resource constraints frequently faced by the eradication programme, planning SIAs on the basis of a statistical model that describes evolving risk is therefore a useful supplement to expert review of epidemiological and vaccination data. Indeed, the statistical model presented here has been used to help plan SIAs in Africa that are described in the GPEI Strategic Plan for 2010–2012 [6]. Furthermore, a 6-mo forecast based on current AFP data can be a helpful aid to the planning of preventive SIAs despite the time taken to analyse the data and the 6-wk lead time required to ensure that sufficient vaccine stocks are available from pharmaceutical companies (personal communication, M. Shirely, United Nations Children's Fund). As an example, AFP data up to 31 December 2010 were available for analysis on 25 January 2011 and were used to predict likely outbreaks from 1 January–30 June 2011. The results from the predictive risk model were communicated to WHO immunisation planners on 16 February 2011.

Our analysis highlights how the geographical risk of poliomyelitis outbreaks has changed over time, particularly in 2010 and 2011, moving from countries surrounding Nigeria to countries bordering those with re-established transmission. This predicted change in risk has been followed by outbreaks in the Congo and Uganda during the period 1 July–31 December 2010. In order to prevent future outbreaks, it is vital that poliovirus transmission is halted in these reinfected countries, as well as in Nigeria. Predictions in 2011 have overestimated the risk in countries south of DRC; with additional longitudinal data the apparent reduced risk may adjust.

Poliovirus exposure estimated from migration and polio incidence accounted for much of the spatial heterogeneity in the number of expected outbreaks per country. In addition, countries bordering Nigeria had a higher number of outbreaks than those not sharing a border. Much of this increased risk will be due to additional local population movement that occurs in addition to that captured by the migration database. We also examined alternative measures of population movement, including international flight and tourism data. However, we found movement that was assumed to be proportional to the number of permanent migrants provided the best fit to the data, presumably because permanent migration is related to broader patterns of cross-border and international travel that is not captured by these other measures. Inclusion of a variable describing changes in exposure to poliovirus over the preceding 18 mo further improved the model fit, presumably capturing aspects of wild-type poliovirus dynamics that are not described by other variables. This may include naturally acquired immunity from the many asymptomatic infections that occur during an outbreak but are not detected using AFP surveillance.

The ability of our study to estimate the impact of routine immunisation and SIAs on population immunity was limited because estimates of routine coverage are known to be measured with error [28], and new methods for surveillance of SIA coverage were only introduced in 2009 [29]. Estimates of trivalent and monovalent OPV effectiveness against poliomyelitis in Nigeria have been used to examine the level and trends in immunity against each poliovirus serotype [13]. Using estimates of immunity derived in this way was not possible here because of the absence of accurate estimates of vaccine effectiveness for most countries in Africa.

An association of <5-y mortality greater than 150 deaths per 1,000 live births with a higher risk of poliomyelitis outbreaks is likely to be indicative of poor sanitary conditions, poverty, high population density, and poor access to health care and nutrition. These factors contribute both to high childhood mortality and poliomyelitis susceptibility [30],[31]. Many countries in Africa have reported an improvement in childhood mortality rates [32], and so susceptibility to outbreaks may be further limited through investment in health care and living conditions.

A limitation of our study is the use of a statistical model that may not capture some of the important non-linearities of poliovirus transmission as eradication is approached. For example, increasing population immunity above a critical vaccination threshold results in herd immunity and could eliminate the risk of polio outbreaks. The log-linear form of the Poisson regression model does not capture these kinds of thresholds. However, no such thresholds were apparent in the data at a country level. The analysis relies on relatively consistent reporting rates for non-polio AFP, as this appears to provide the best estimate of population immunity. Unfortunately, not all countries reported a consistently high AFP rate, which will introduce uncertainties in the analysis. In addition, estimates of human migration and poliovirus exposure across Africa were shown to be associated with polio outbreaks, but there is likely to be much temporal variation in human movement patterns that was not captured in the available data. This could explain why the magnitudes of the polio outbreaks in mid-2004 and mid-2008 were not fully captured by the model, and why the westward spread of polio from Nigeria in 2008 and 2009 was not completely replicated in the model. Other sources of movement patterns within Africa are required to fully capture this important determinant of disease transmission.

Countries bordering Nigeria experienced more frequent outbreaks, but in general these were reduced in duration and size compared with outbreaks in other countries in Africa. This association could be explained by unmeasured increases in population immunity following exposure to wild-type poliovirus. If natural exposure to poliovirus is the cause of the reduced risk, outbreak size in these countries should be carefully monitored as the incidence in Nigeria reduces. Serological surveys for antibodies to poliovirus within populations in Africa would also supplement current surveillance for AFP and poliovirus in understanding polio epidemiology.

Although there is always an element of uncertainty and chance in the distribution of infectious disease outbreaks, this study highlights that poliomyelitis outbreaks in Africa are largely governed by the extent of immunity in the population, population movement, and exposure to infection. Planning SIA campaigns based on evolving risk may reduce the number of outbreaks by responding to increased risk prior to an outbreak occurring. As the incidence of polio in Nigeria has remained very low in 2010 and 2011, there may be a unique opportunity to eliminate polio from Africa in the near-term through targeted vaccination informed by appropriate predictive models.

## Supporting Information

Text S1 **Supplementary methods and results.**
(DOC)

## Abbreviations

AIC: Akaike's information criteria
AFP: acute flaccid paralysis
AUC: area under the curve
CI: confidence interval
DRC: the Democratic Republic of the Congo
GPEI: Global Polio Eradication Initiative
OPV: oral poliovirus vaccine
SE: standard error
SIA: supplementary immunisation activity
WHO: World Health Organization
