# Supplementary material for: A Statistical Model of the International Spread of Wild Poliovirus in Africa Used to Predict and Prevent Outbreaks
Source: PLoS Med. 2011 Oct 18;8(10):e1001109. doi: 10.1371/journal.pmed.1001109 (PMC3196484; doi:10.1371/journal.pmed.1001109)

**Supplementary information (S1) for “Use of real-time surveillance data to predict and prevent international spread of wild poliovirus in Africa” by O’Reilly et al.**

**Additional notes to the Methods**

Table 1. Summary of the number of supplementary immunisation activities (SIAs), the proportion that were national and comments regarding whether the country should be split into regions according to the previous SIA strategies in that country.

| **Country** | **Number of SIAs 2003-2009** | **Proportion of SIAs that were national** | **Comments about necessity to partition country** |
| --- | --- | --- | --- |
| **Sudan** | 78 | 0.38 | **Partitioned** |
| **Somalia** | 57 | 0.37 | Many of the districts were missed due to inaccessibility and in response to outbreaks in neighbouring Ethiopia |
| **Chad** | 48 | 0.5 | A majority of SIAs were carried out in response to outbreaks already present. OPV doses from AFP data do not vary regionally |
| **Ethiopia** | 44 | 0.25 | **Partitioned** |
| **Niger** | 41 | 0.56 | A majority of SIAs were carried out in response to outbreaks already present. Some northern areas have been missed in SIAs. OPV doses from AFP data does not vary regionally |
| **Democratic Republic of the Congo** | 40 | 0 | **Partitioned** |
| **Angola** | 29 | 0.72 | A majority of SIAs were carried out in response to outbreaks already present. OPV doses from AFP data does not vary regionally |
| **Cameroon** | 23 | 0.39 | A majority of SIAs were carried out in response to outbreaks already present. OPV doses from AFP data does not vary regionally |
| **Benin** | 22 | 0.82 | Too few SIAs to warrant partition, and majority of SIAs are national |
| **Burkina faso** | 21 | 0.86 | Too few SIAs to warrant partition, and majority of SIAs are national |
| **Central African Republic** | 21 | 0.9 | Too few SIAs to warrant partition, and majority of SIAs are national |
| **Ivory Coast** | 20 | 1 | All national, no need to divide |
| **Republic of the Congo** | 18 | 0.56 | Too few SIAs to warrant partition |
| **Mali** | 18 | 0.86 | Too few SIAs to warrant partition, and majority of SIAs are national |
| **Ghana** | 17 | 0.88 | Too few SIAs to warrant partition, and majority of SIAs are national |
| **Namibia** | 16 | 0.88 | Too few SIAs to warrant partition, and majority of SIAs are national |
| **Togo** | 15 | 1 | All national, no need to divide |
| **Uganda** | 12 | 0.17 | Too few SIAs to warrant partition |
| **Guinea** | 12 | 1 | All national, no need to divide |
| **Kenya** | 11 | 0 | Majority were in response to outbreak in Kenya |
| **Liberia** | 10 | 0.92 | Too few SIAs to warrant partition, and majority of SIAs are national |
| **Sierra Leone** | 10 | 1 | All national, no need to divide |
| **Algeria** | 8 | 0 | Too few SIAs to warrant partition |
| **South Africa** | 7 | 0.71 | Too few SIAs to warrant partition |
| **Equatorial Guinea** | 7 | 1 | All national, no need to divide |
| **Mauritiana** | 6 | 1 | All national, no need to divide |
| **Libya** | 5 | 0.4 | too few SIAs to warrant partition, and majority of SIAs are national |
| **Eritrea** | 5 | 0.8 | Too few SIAs to warrant partition, and majority of SIAs are national |
| **Gambia** | 5 | 0.8 | Too few SIAs to warrant partition, and majority of SIAs are national |
| **Cape Verde** | 4 | 1 | All national, no need to divide |
| **Gabon** | 4 | 1 | All national, no need to divide |
| **Guinea Bissau** | 4 | 1 | All national, no need to divide |
| **Senegal** | 4 | 1 | All national, no need to divide |
| **Swaziland** | 4 | 1 | All national, no need to divide |
| **Burundi** | 3 | 0 | Too few SIAs to warrant partition |
| **Mozambique** | 3 | 0 | Too few SIAs to warrant partition |
| **Egypt** | 3 | 0.33 | Too few SIAs to warrant partition |
| **Sao Tome and Principe** | 3 | 1 | All national, no need to divide |
| **Comoros Islands** | 2 | 0 | Too few SIAs to warrant partition |
| **Rwanda** | 2 | 0 | Too few SIAs to warrant partition |
| **Botswana** | 2 | 1 | All national, no need to divide |
| **Djibouti** | 2 | 1 | All national, no need to divide |
| **Lesotho** | 2 | 1 | All national, no need to divide |
| **Tanazania** | 1 | 0 | too few SIAs to warrant partition |
| **Zimbabwe** | 1 | 1 | All national, no need to divide |
| **Madagascar** | 0 | na |  |
| **Malawi** | 0 | na |  |
| **Zambia** | 0 | na |  |
| **Mauritius** | 0 | na |  |
| **Morrocco** | 0 | na |  |

For most of the countries in the dataset, the non-polio AFP data were split into 6 month intervals to explore associations between vaccine-induced population immunity and the number of observed poliomyelitis outbreaks. For Ethiopia, Democratic republic of the Congo (DRC), and Sudan, all of which are large countries, the population immunity varies regionally, reflecting the regional SIAs carried out according to the perceived difference in outbreak risk. To better reflect these differences, these countries were each split into three regions in the analysis (Figure S1). For all countries, the variance of the reported vaccine-induced immunity was less when the country was partitioned when compared to the country-wide variance of reported immunity (Table S1).

**Figure S1: Regions of Ethiopia, Sudan and Democratic Republic of the Congo analysed separately in analysis of poliomyelitis outbreaks.**

Table S2: Mean and variance of the proportion of non-polio AFP children reporting three or more doses in the previous 6 months at a country and regional level.

| Country / region | Description | mean value | variance |
| --- | --- | --- | --- |
| **SUD** | **Sudan** | **0.771** | **0.053** |
| SUD3 | Darfur | 0.925 | 0.006 |
| SUD1 | Northern states | 0.649 | 0.008 |
| SUD2 | Southern states | 0.887 | 0.009 |
| **RDC** | **Democratic republic of the Congo (DRC)** | **0.639** | **0.035** |
| RDC1 | Bas-Congo, Kasai-Occidental, Bandundu | 0.738 | 0.006 |
| RDC2 | Equateur, Province Oriental, N-Kivu, S-Kivu | 0.686 | 0.004 |
| RDC3 | Rest of DRC | 0.657 | 0.003 |
| **ETH** | **Ethiopia** | **0.622** | **0.038** |
| ETH1 | Tigray, Amhara | 0.763 | 0.011 |
| ETH2 | Somali, afar and 2 districts in Oromia (E & W Herenge | 0.436 | 0.017 |
| ETH3 | Rest of Ethiopia | 0.682 | 0.006 |

**Description of approximations of poliomyelitis exposure**

To account for poliomyelitis exposure from countries and regions reporting cases, several approximations were explored. Data on international migration, tourism (from Nigeria only) and flight data (from Nigeria only) were used to calculate poliomyelitis exposure (*λ*);

$\lambda_{j,t}=\sum_{i=1}^{n} m_{i,j}x_{i,t-1}$,

where $m_{i,j}$ is the reported number of individuals in each dataset who moved from *i* to *j*, $x_{i,t-1}$ is the number of poliomyelitis cases reported in country *i* during the preceding six month period and *n* is the number of countries. In other words, the incidence in country *i* at *t*-1 was multiplied by the reported number of movements from *i* to *j*, and this value was summed for all countries in Africa to obtain the total poliovirus exposure in country *j*. When calculating the poliomyelitis exposure from only Nigeria (for all approximations), n=1.

Gravity models, where population movement is approximated through the product of the population size in *i* and *j* and divided by the distance ($d_{ij}$) between the two locations;

$$m_{ij}=\frac{p_{i}p_{j}}{d_{ij}}$$

The value of $m_{ij}$ is then used in the equation for calculating poliomyelitis exposure. A high value of $m_{ij}$will be obtained when there are large population sizes with little distance between them, smaller values of $m_{ij}$ will occur with small population size and large distances between them. This is the simplest version of a gravity model, powers may also be included and estimated for each term in the equation, however this is not possible in the current analysis. Gravity models have been shown to approximate well the heterogeneous contact between cities and resultant influenza and measles epidemics (*Xia Y, Bjornstad ON, Grenfell BT (2004) Measles metapopulation dynamics: a gravity model for epidemiological coupling and dynamics. Am Nat 164: 267-281* and *Eggo RM, Cauchemez S, Ferguson NM Spatial dynamics of the 1918 influenza pandemic in England, Wales and the United States. J R Soc Interface 8: 233-243*).

**Results**

**Table 3. Summary of the number of outbreaks experienced by each country, and average values for the non-polio AFP rate, routine coverage and percentage of children reporting three or more OPV doses in Africa.**

| **Country** | **Freq.** | **mean size** | **Dates reported for first case of an outbreak** | **Average non-polio AFP rate^1^** | **Average routine coverage** | **Average percentage of non-polio AFP reporting three or more doses** | **Average value of poliovirus exposure (logged)** |
| --- | --- | --- | --- | --- | --- | --- | --- |
| **Algeria** | 0 | na | na | 0.31 | 91.8 | 90.5 | 12.6 |
| **Angola** | 3 | 28 | 2005, 2007, 2008 | 1.82 | 63.1 | 56.4 | 10.8 |
| **Benin** | 7 | 5 | 2003.5, 2008-2009 | 1.52 | 69.9 | 82.7 | 15.6 |
| **Botswana** | 1 | 1 | 2004 | 1.24 | 82.7 | 94.8 | 16 |
| **Burkina Faso** | 5 | 8 | 2003, 2008.5, 2009 | 1.85 | 96 | 84.2 | 10.4 |
| **Burundi** | 1 | 2 | 2009.5 | 0.97 | 87.6 | 93.6 | 14.5 |
| **CAF** | 5 | 10 | 2003.5-2004.5, 2008, 2009 | 1.37 | 78.1 | 74 | 16.7 |
| **Cameroon** | 13 | 2 | 2003.5, 2004.5-2005, 2006.5, 2009.5 | 3.86 | 46.5 | 76.5 | 13.5 |
| **Cape Verde** | 0 | na | na | 3.37 | 97 | 100 | 9.1 |
| **Chad** | 14 | 14 | various years | 1.54 | 36 | 61.8 | 14.7 |
| **Congo** | 1 | 50 | 2010.5 | 1.64 | 74.9 | 80 | 15.7 |
| **Cote dIvoire** | 3 | 15 | 2003.5, 2008.5-2009 | 2.25 | 78.3 | 73.9 | 12.9 |
| **Comoros** | 0 | na | na | 2.8 | 81 | 66.7 | 10 |
| **Djibouti** | 0 | na | na | 1.12 | 79.5 | 75 | 13.5 |
| **Egypt** | 0 | na | na | 2.67 | 97.5 | 98.5 | 12.4 |
| **Equatorial Guinea** | 0 | na | na | 1.86 | 39 | 67.4 | 11.3 |
| **Eritrea** | 1 | 1 | 2005 | 1.35 | 96 | 91 | 12.8 |
| **Ethiopia** | 4 | 11 | 2004.5, 2005.5, 2008 | 0.35 | 70.6 | 76.9 | 13.9 |
|  |  |  |  | 0.17 | 70.6 | 43.1 | 12.6 |
|  |  |  |  | 0.7 | 70.6 | 69.1 | 14.6 |
| **Gabon** | 0 | na | na | 1.78 | 44 | 65.6 | 15.1 |
| **Gambia** | 0 | na | na | 1.3 | 94.8 | 100 | 11.6 |
| **Ghana** | 3 | 5 | 2003, 2008.5 | 1.35 | 85.6 | 92.7 | 16.4 |
| **Guinea bissau** | 0 | na | na | 0.91 | 61.8 | 88.3 | 10.3 |
| **Guinea** | 2 | 27 | 2004.5, 2009 | 1.22 | 70.9 | 66.6 | 15.8 |
| **Kenya** | 2 | 11 | 2006.5, 2009 | 0.9 | 73.3 | 78.2 | 15.8 |
| **Lesotho** | 0 | na | na | 1.2 | 84.2 | 96.7 | 8.8 |
| **Liberia** | 2 | 8 | 2009, 2010 | 2.79 | 70 | 68.5 | 14.2 |
| **Lybia** | 0 | na | na | 1.28 | 97.6 | 98 | 13.1 |
| **Madagascar** | 0 | na | na | 0.83 | 74.5 | 83 | 9.3 |
| **Mali** | 10 | 3 | 2004-2004.5, 2008.5-2010.5 | 0.97 | 73.9 | 82.8 | 13.2 |
| **Malawi** | 0 | na | na | 0.55 | 92.7 | 89.9 | 11.9 |
| **MAS** | 0 | na | na | 1.21 | 97.8 | 95.8 | 7.8 |
| **Mauritania** | 1 | 18 | 2009.5 | 1.88 | 69.2 | 88.9 | 10.9 |
| **Morocco** | 0 | na | na | 0.5 | 97.3 | 82.7 | 11.9 |
| **Mozambique** | 0 | na | na | 0.55 | 75 | 87.5 | 12.1 |
| **Namibia** | 1 | 19 | 2006 | 1.88 | 80.9 | 92.2 | 12.5 |
| **Niger** | 33 | 2 | 2005-2010 | 2.33 | 57.5 | 77.8 | 16 |
| **DRCongo** | 7 | 9 | 2006-2006.5, 208.5-2009 | 0.6 | 63.7 | 73.3 | 13.9 |
|  |  |  |  | 1.03 | 63.7 | 68.8 | 14.8 |
|  |  |  |  | 0.88 | 63.7 | 65.3 | 14.2 |
| **Rwanda** | 0 | na | na | 1.45 | 96 | 94.1 | 11.3 |
| **Senegal** | 3 | 6 | 2010 | 1.59 | 85.7 | 85 | 13.3 |
| **Sierra Leone** | 1 | 12 | 2009.5 | 2.3 | 68.7 | 75.8 | 12.6 |
| **South Africa** | 0 | na | na | 0.75 | 70 | 90.3 | 14.4 |
| **Somalia** | 1 | 228 | 2005.5 | 2.67 | 31.9 | 60.1 | 13.2 |
| **Sao Tome** | 0 | na | na | 2.01 | 99 | 100 | 11.1 |
| **Sudan** | 8 | 28 | 2004-2004.5, 2007.5-2008.5 | 1.11 | 80.1 | 93.1 | 16.3 |
|  |  |  |  | 0.56 | 80.1 | 67.4 | 17.4 |
|  |  |  |  | 0.18 | 80.1 | 89.8 | 16.4 |
| **Swaziland** | 0 | na | na | 1.66 | 95.7 | 95.8 | 11.3 |
| **Tanzania** | 0 | na | na | 0.86 | 90.7 | 90 | 12 |
| **Togo** | 4 | 3 | 2003.5, 2008.5-2009 | 1.63 | 82.4 | 85 | 15.3 |
| **Uganda** | 2 | 6 | 2009, 2010.5 | 1.31 | 0 | 80.1 | 13.8 |
| **Zambia** | 0 | na | na | 1.19 | 0 | 83.6 | 13.7 |
| **Zimbabwe** | 0 | na | na | 0.68 | 0 | 89.1 | 13.9 |

^1^Please not that the values of the non-polio AFP rate will be slightly lower than that reported elsewhere (<http://apps.who.int/immunization_monitoring/en/diseases/poliomyelitis>) as additional requirements are included in the current analysis (AFP cases considered additionally include children under the age of 5 (therefore either the date of birth or age must be specified), rather than children being under 15 years of age).


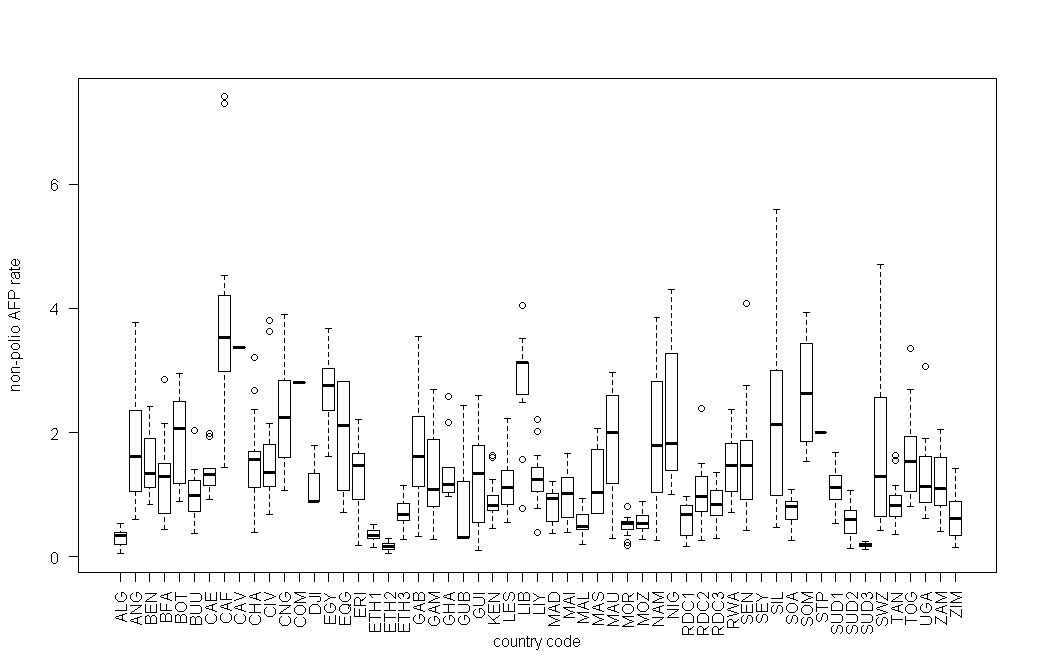


Figure 2. Box-plot of the distribution of the non-polio AFP rate by country from 2003-2010. Each box-plot specifies the median (middle bar) and 25-75^th^ percentile values (boxes) of the non-polio AFP rate for each country.

Table 4. Average country specific non-polio AFP rate in children under 5 years of age in Africa. Mean value of the non-polio AFP rate illustrates that from 2003 to 2005 there was a gradual increase in the non-polio AFP rate from below 1.00 to above 1.00. After 2005 the AFP rate has remained above 1.00, with some additional increase. The variance of the non-polio AFP rate has remained relatively constant.

| **Six month time period** | **Relative non-polio AFP rate (under 5's)** | **Variance per six month period** |
| --- | --- | --- |
| **2003.5** | 0.88 | 0.38 |
| **2004** | 0.90 | 0.53 |
| **2004.5** | 0.96 | 0.40 |
| **2005** | 0.99 | 0.34 |
| **2005.5** | 1.58 | 1.71 |
| **2006** | 1.56 | 1.76 |
| **2006.5** | 1.36 | 0.78 |
| **2007** | 1.33 | 0.79 |
| **2007.5** | 1.51 | 0.83 |
| **2008** | 1.61 | 1.18 |
| **2008.5** | 1.60 | 0.95 |
| **2009** | 1.79 | 1.20 |
| **2009.5** | 1.21 | 0.93 |
| **2010** | 1.64 | 1.43 |
| **2010.5** | 0.94 | 0.59 |

Table S3: Correlation coefficients of variables describing immunity profile of countries in Africa from 2003-2009. **All coefficients were significant at the *P*<0.001 level, using a two-tailed test for determining statistical significance.**

| Variable | thr.doses_6 | med.doses_6 | mean.doses_6 | rou.cov | SIA_6 |
| --- | --- | --- | --- | --- | --- |
| zero.doses_6 | -0.630 | -0.244 | -0.249 | -0.271 | +0.153 |
| SIA_6 | -0.227 | +0.196 | +0.239 | -0.227 | -- |
| rou.cov | +0.537 | +0.272 | +0.244 | -- |  |
| mean.doses_6 | +0.397 | +0.957 | -- |  |  |
| med.doses_6 | +0.400 | -- |  |  |  |

Table S4. Variables in the Poisson mixed effects model that were associated with the number of poliomyelitis outbreaks in Africa from 2003 to 2010 (n=622 from 56 countries, AIC=553.82). The proportion of children reporting 3 or more doses of OPV was replaced with the WHO/UN estimate of routine coverage.

|  |  | | |  | **std. dev. of random effect** |
| --- | --- | --- | --- | --- | --- |
| **Description of risk factor** | **Incidence risk ratio (IRR, 95% CI)** | | | **p-value** |
| poliomyelitis exposure from all African countries - 1 unit increase (when logged) | 1.34 | 1.16 | 1.54 | <0.001 | na |
| poliomyelitis exposure in previous six months was higher than exposure 18 months ago (vs. lower) | 2.00 | 1.33 | 3.00 | 0.002 | na |
|  |  |  |  |  |  |
| Country borders Nigeria (vs. no) | 6.09 | 2.87 | 12.92 | <0.001 | na |
| Routine coverage (10% increase) | 1.00 | 1.00 | 1.00 | 0.659 | 0.71 |
| Country reported an under 5 mortality rate greater than 150 deaths per 1000 at-risk population (vs. lower) | 2.77 | 1.55 | 4.98 | 0.001 | na |

Table S5. Area under the curve values (AUC) for regression model and comparison of using historical data as a predictor for 6 month ahead projections.

| Dataset | Categories used | Area under the curve (AUC) | Standard error (SE) |
| --- | --- | --- | --- |
| Regression model | 40 | 0.82 | 0.03 |
| Predictor being an outbreak in last two time points | 40 | 0.77 | 0.05  (poor sensitivity) |
| Predictor being an outbreak in last three time points | 40 | 0.73 | 0.05  (poor sensitivity) |
| Predictor being an outbreak in last four time points | 40 | 0.73 | 0.05  (poor sensitivity) |
| Predictor being an outbreak in last five time points | 40 | 0.74 | 0.05  (poor sensitivity) |
| Predictor being an outbreak in last six time points | 40 | 0.76 | 0.05  (poor sensitivity) |

**Table S6. Variables tested in the Cox proportional hazards model exploring factors associated with an increased duration of a multiple-case outbreak and the log-likelihood values in univariable analysis.** Explanatory variables that were significantly (*p*<0.2) associated with an increased duration of an outbreak were tested in a multivariable model.

| **Variable** | **Description** | **Estimate** | **p-value** | **Log-likelihood** |
| --- | --- | --- | --- | --- |
| **thrdoses_6** | Percentage of non-polio AFP cases under 5 years reporting three or doses of OPV in the previous 6 months | 3.80 | 0.007 | -535.53 |
| **afp5_6rel** | relative AFP rate in the previous 6 months | 1.25 | 0.004 | -535.73 |
| **borderend** | Country borders Nigeria (0/1) | 1.63 | 0.006 | -535.86 |
| **meddoses_6** | Median number of OPV doses reported in non-polio AFP under 5 years in the previous 6 months | 1.17 | 0.004 | -535.88 |
| **meandoses_6** | Mean number of OPV doses reported in non-polio AFP under 5 years in the previous 6 months | 1.17 | 0.004 | -535.94 |
| **afprel_2** | relative AFP rate in the previous 6 months greater than 2.0 | 1.54 | 0.031 | -537.40 |
| **roucov** | routine coverage | 1.01 | 0.063 | -537.74 |
| **firstsia2** | Time between first case and next SIA in country | 1.00 | 0.148 | -538.41 |
| **density** | population density | 1.00 | 0.120 | -538.51 |
| **u5mortb** | incidence of childhood mortality in under 5's greater than 150 per 1000 live births (vs. lower) | 0.78 | 0.171 | -538.65 |
| **popprop014** | population size of 0-14 year olds (logged) | 1.01 | 0.289 | -538.92 |
| **zerodoses_6** | Percentage of non-polio AFP cases under 5 years reporting zero doses of OPV in the previous 6 months | 0.27 | 0.309 | -539.02 |
| **sia6** | Number SIAs in the first six months after the start of the outbreak | 0.96 | 0.329 | -539.08 |
| **u5mort** | incidence of childhood mortality in under 5's per 1000 live births (crude estimate) | 1.00 | 0.450 | -539.29 |
| **sia1** | Number SIAs in the first month after the start of the outbreak | 0.95 | 0.707 | -539.50 |
| **sia2** | Number SIAs in the first two months after the start of the outbreak | 1.03 | 0.746 | -539.52 |
| **sia3** | Number SIAs in the first three months after the start of the outbreak | 0.98 | 0.805 | -539.54 |

**Table S7. Variables tested in the censored negative binomial model exploring factors associated with an increased size of multiple-case outbreaks and the log-likelihood values in univariabl**e analysis.

| Variable | Description | Estimate | p-value | Log-likelihood |
| --- | --- | --- | --- | --- |
| thrdoses_6 | Proportion of non-polio AFP cases under 5 years reporting three or doses of OPV in the previous 6 months | -5.60537 | <0.001 | -273.329 |
| borderend | Country borders Nigeria (0/1) | -1.69481 | <0.001 | -275.884 |
| popprop014 | population size of 0-14 year olds (logged) | -0.15104 | 0.004 | -286.98 |
| roucov | routine cov | -0.02028 | 0.027 | -288.827 |
| meddoses_6 | Median number of OPV doses reported in non-polio AFP under 5 years in the previous 6 months | -0.24819 | 0.025 | -288.96 |
| tOPV_6 | Number of tOPV SIAs in the previous 6 months | -0.2308 | 0.026 | -289.118 |
| firstsia2 | Time between first case and next SIA in country | 0.010305 | 0.071 | -289.303 |
| meandoses_6 | Mean number of OPV doses reported in non-polio AFP under 5 years in the previous 6 months | -0.16424 | 0.133 | -290.287 |
| pops014l | population size (logged) | 0.151489 | 0.411 | -291.042 |
| afp5_6rel | relative AFP rate in the previous 6 months | 0.20943 | 0.616 | -291.252 |

Figure S2. ROC curve of predictive ability of the model to identify countries likely to experience an outbreak in the next six months (grey area is the 95^th^ percentile confidence intervals of the parametric model). The estimate of the number of outbreaks was a continuous variable and thus was divided into 20 groups. To generate a receiver-operator characteristic (ROC) curve, cut-off values for the predicted number of outbreaks are used to estimate the model sensitivity and specificity at these cut-offs. The sensitivity of the model describes the probability that the predicted number of outbreaks per country is above each cut-off given that an outbreak was subsequently observed. The specificity is the probability that the predicted number of outbreaks was below each cut-off given that no outbreak was observed. 1-Specificity is the false positive rate. An ROC plot allows graphical assessment of how well a test, in this case a regression model, performs in detection of an output of interest, in this case predicting an outbreak within a country. The binormal parametric model extends this graphical assessment to estimation of the area under the curve, which provides an overall assessment of the predictive ability of the model.

**Figure S3. Kaplan-Meier curves for the time to the last case of an outbreak for the three variables in the multivariable survival model.** (A) Outbreaks in countries bordering Nigeria are plotted separately to outbreaks in countries not bordering Nigeria. (B) Outbreaks where the percentage of non-polio AFP children reporting three or more doses of AFP was greater than 80% are plotted separately to those reporting less than 80%. For each figure censored outbreaks are denoted by the tick on the Kaplan-Meier curve.


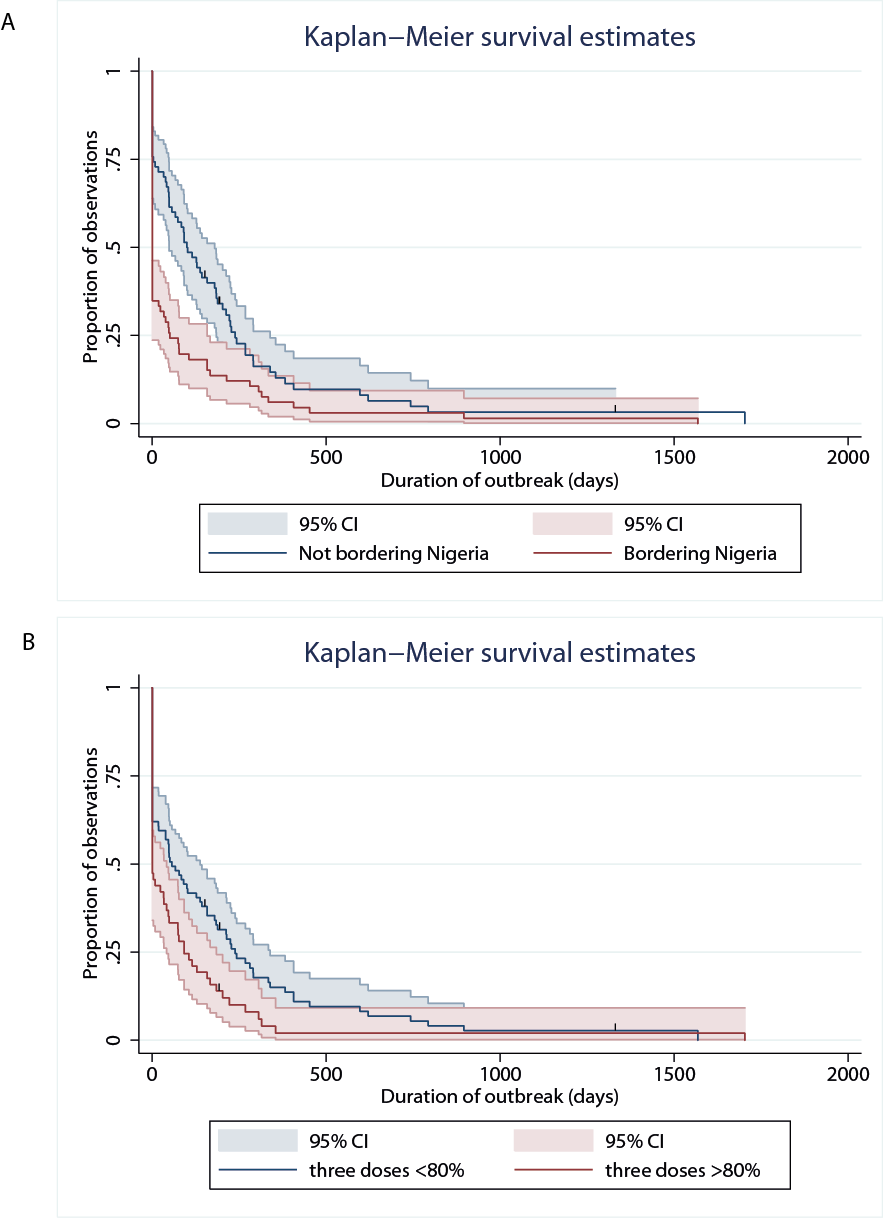

Supplement: Text S1 — Supplementary methods and results. (DOC) [file pmed.1001109.s001.doc]
